# Placental Abruption Complicated by the Couvelaire Uterus: A High-Risk Obstetric Case at 30 Weeks Gestation

**DOI:** 10.7759/cureus.46832

**Published:** 2023-10-11

**Authors:** Garapati Jyotsna, Surekha Tayade, Sakshi Sharma, Drashti Patel, Sukanya Singh

**Affiliations:** 1 Obstetrics and Gynaecology, Jawaharlal Nehru Medical College, Datta Meghe Institute of Higher Education and Research, Wardha, IND

**Keywords:** prenatal care awareness, multidisciplinary approach, maternal-fetal outcomes, obstetric emergency, couvelaire uterus, placental abruption

## Abstract

Placental abruption, a rare but life-threatening obstetric emergency, presents substantial risks to maternal and fetal well-being. This case report documents the clinical journey of a 35-year-old woman with multiple risk factors who presented at 30 weeks gestation with symptoms suggestive of placental abruption, including colicky lower abdominal pain and vaginal bleeding. Notably, her late initiation of prenatal care and a history of pregnancy-induced hypertension added complexity to the clinical picture. The case revealed a Couvelaire uterus, an uncommon and challenging complication of placental abruption, further emphasizing the need for early recognition and swift intervention. A multidisciplinary approach played a pivotal role in managing this high-risk obstetric case. Imaging and laboratory tests facilitated diagnosis and assessment, guiding surgical intervention and post-operative care. Despite the severity of the condition, the patient experienced a positive outcome for herself and her fetus, highlighting the critical importance of timely and comprehensive medical care. This case report contributes to medical knowledge by shedding light on the rare Couvelaire uterus. It underscores the significance of early diagnosis, coordinated healthcare teams, and patient education in mitigating risks associated with placental abruption. Ultimately, it reinforces the vital role of healthcare providers in safeguarding the lives of expectant mothers and their infants in obstetric emergencies.

## Introduction

Placental abruption, an infrequent yet perilous obstetric emergency, poses substantial threats to the well-being of both mother and fetus [1]. This case report aims to provide a comprehensive overview of placental abruption, including its clinical manifestations, complications, and outcomes. Additionally, we will delve into the unique and rare presentation of a Couvelaire uterus, a severe manifestation of placental abruption.

Placental abruption is a critical obstetric complication characterised by prematurely separating the placenta from the uterine wall before the delivery of the fetus. This condition can lead to significant maternal and fetal morbidity and mortality if not promptly recognised and managed. Therefore, it is essential to understand the signs, symptoms, and potential complications associated with this condition [2].

Abruptio placenta typically presents symptoms such as colicky lower abdominal pain and vaginal bleeding. Recognising these clinical manifestations promptly is crucial, as they often necessitate immediate medical attention. Moreover, understanding the gestational age at which placental abruption occurs can significantly impact maternal and fetal outcomes. Therefore, this case report will explore the gestational age differences and their implications [3].

Placental abruption, defined as the premature detachment of the placenta from the uterine wall after 20 weeks of gestation, represents a condition fraught with grave consequences for both the expectant mother and the developing fetus [2]. Its clinical presentation can exhibit considerable variation, and the extent of the abruption may only sometimes be apparent. A Couvelaire uterus, characterised by extensive infiltration of blood into the uterine myometrium, represents an exceedingly uncommon and intricate complication of placental abruption. Timely recognition and swift intervention are paramount in effectively managing this condition [3].

This case report offers a comprehensive account of the patient's clinical journey, encompassing her initial presentation with alarming symptoms and deteriorating vital signs, culminating in surgical exploration that resulted in the delivery of the fetus and placenta. Additionally, we delve into the patient's post-operative recovery, emphasising the importance of a multidisciplinary approach in addressing the intricate interplay of factors in cases of severe placental abruption. The favourable outcome underscores the significance of early diagnosis, prompt surgical intervention, and vigilant post-operative care, emphasising the pivotal role of healthcare professionals in handling such high-stakes obstetric scenarios [4].

## Case presentation

A 35-year-old woman, gravida three para one, presented to the emergency department during the late night hours when she was at 30 weeks gestation in her second pregnancy. She complained of colicky lower abdominal pain and vaginal bleeding, which had been ongoing for six hours. During the history-taking process, it was revealed that she had previously used one sanitary pad, which was only mildly soaked. Her medical history included one full-term normal vaginal delivery complicated by pregnancy-induced hypertension one year earlier. Notably, she had not received prenatal care at our hospital and was only booked for care at 21 weeks in a private facility. Until this point, she had been relatively asymptomatic, with two antenatal visits. It's noteworthy that she declined maternal serum screening for chromosomal and aneuploidies. Additionally, a fetal anomaly screening at 21 weeks had shown no signs of fetal anomalies.

Upon examination, the patient's vital signs were concerning, with maternal tachycardia of 110 beats per minute and a blood pressure reading of 90/70 mmHg. Abdominal examination revealed a contracted and tense abdomen that felt woody-hard. A speculum examination showed active bleeding through the cervical os, and a small clot was evacuated during the examination.

Subsequently, the patient was admitted to the hospital for further monitoring. Initial blood tests revealed a haemoglobin level of 8.4 g/dL, a low platelet count of 90,000/cumm, and a deranged coagulation profile characterized by prolonged prothrombin time (PT). They activated partial thromboplastin time (aPTT). An abdominal ultrasound showed a heterogeneous area anterior to the placenta, suggesting blood products, indicating placental abruption.

Given the patient's deteriorating condition and the need to explore the unique aspects of this case, we opted for an explorative laparotomy. Under general anaesthesia, a lower segment hysterotomy was performed via a Pfannenstiel incision. This approach allowed us to gain crucial insights into the condition of the patient's uterus and the complications arising from the Couvelaire uterus, a relatively rare and severe manifestation of placental abruption.

**Figure 1:**
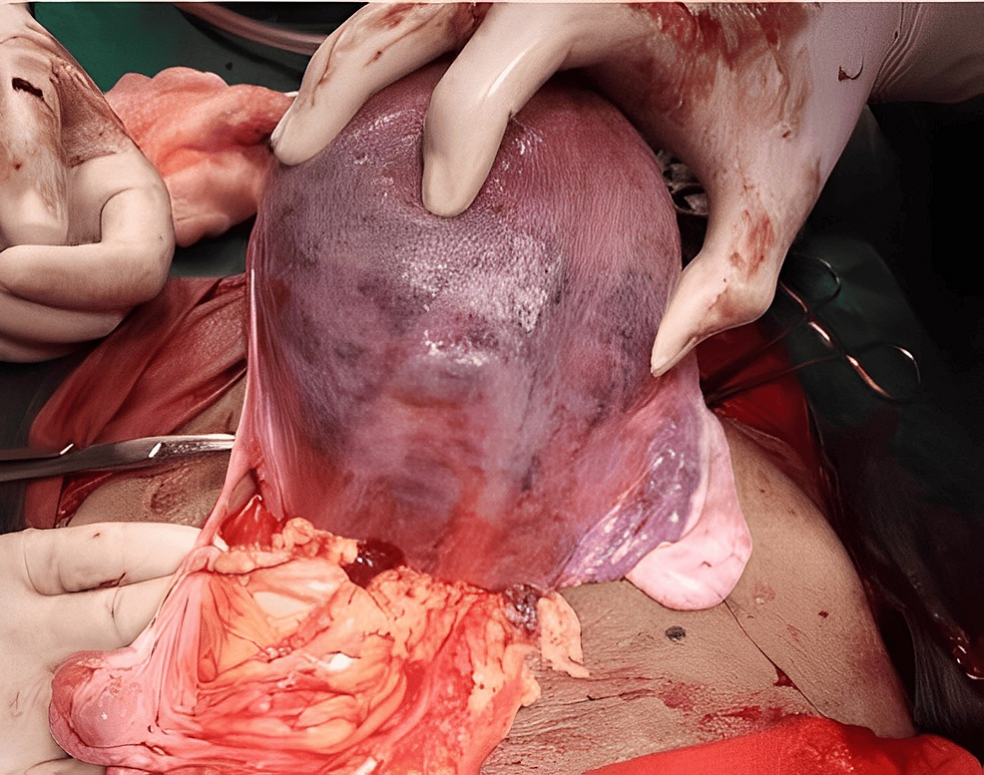
The dark purple and copper colour patches with ecchymosis and indurations diagnostic of Couvelaire uterus or uteroplacental apoplexy - posterior view

**Figure 2:**
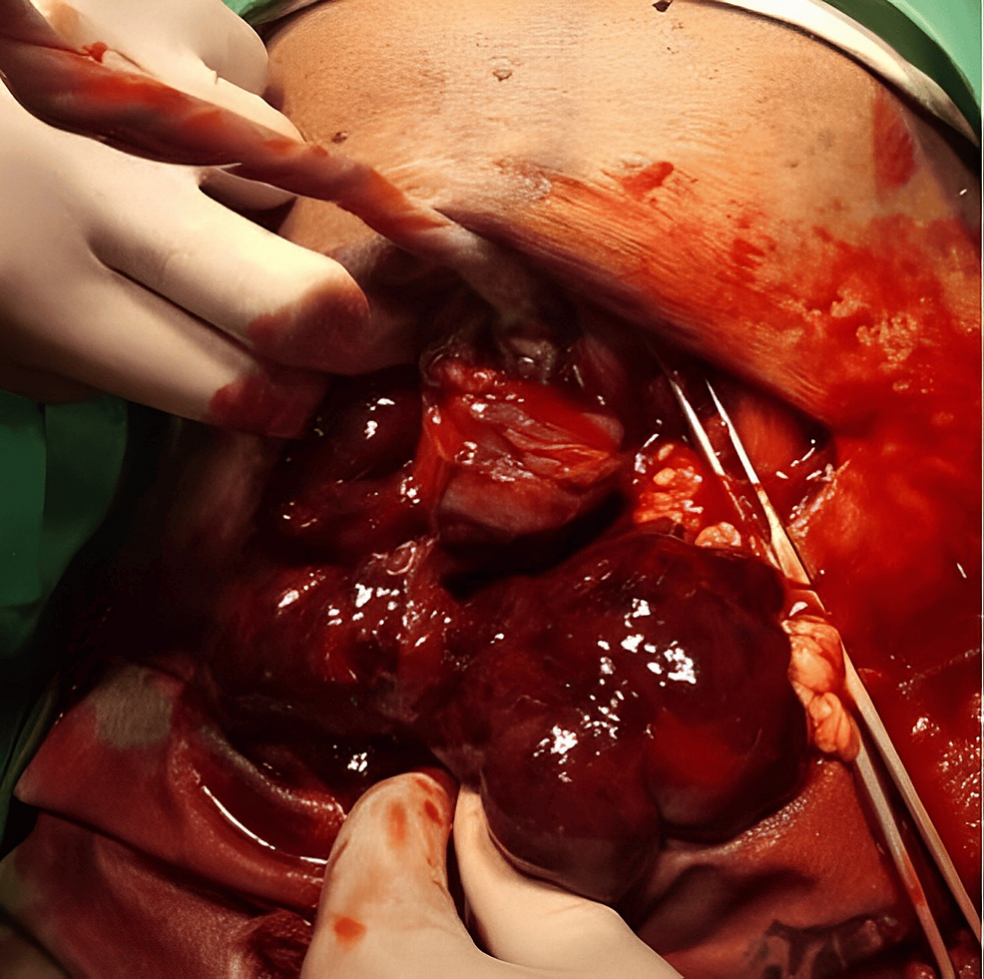
Blood and blood clots noted on incision into the uterus and after delivery of the fetus

During the laparotomy, our observations unveiled several noteworthy findings. Notably, we encountered a Couvelaire uterus (Figure 1), a condition marked by the infiltration of blood into the myometrium due to placental abruption. Upon making an incision into the uterus (Figure 2), we observed the presence of clots, further confirming the diagnosis of a Couvelaire uterus.

**Figure 3:**
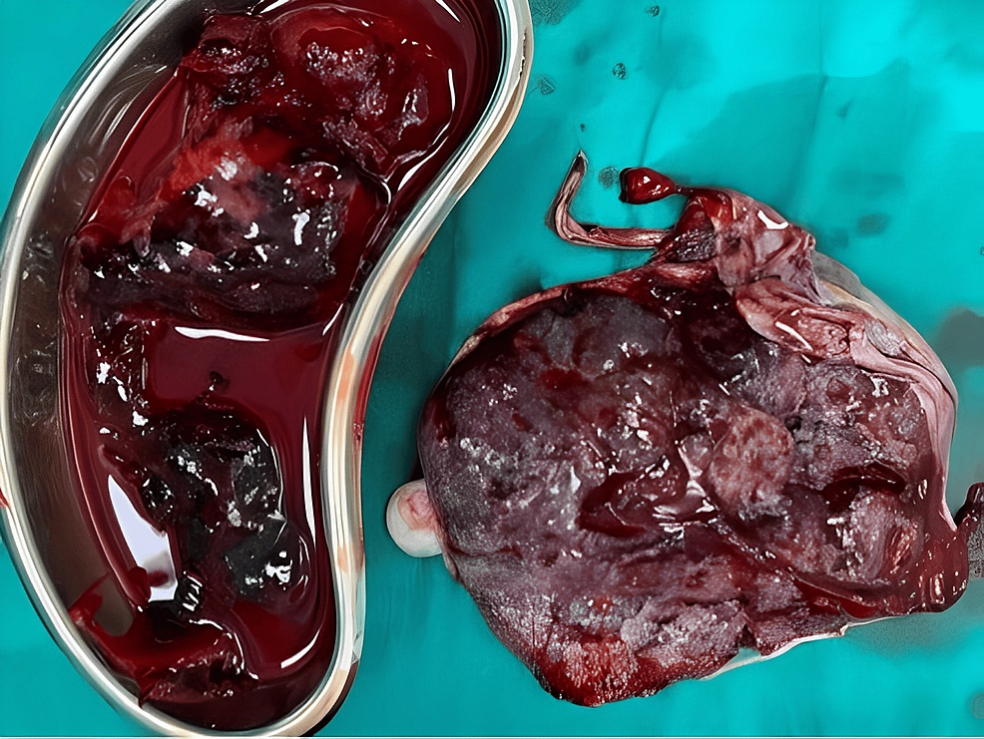
Estimated blood loss was 800 ml with larger blood clots

As we proceeded with the surgery, we encountered the fetus and an entirely separated placenta. One of the major complications associated with a Couvelaire uterus is hemoperitoneum, a condition characterised by blood accumulating within the abdominal cavity. Unfortunately, this case presented with hemoperitoneum, which was evident during the procedure. The blood loss is approximately 800 mL (Figure 3), emphasising the severity of this complication.

**Figure 4:**
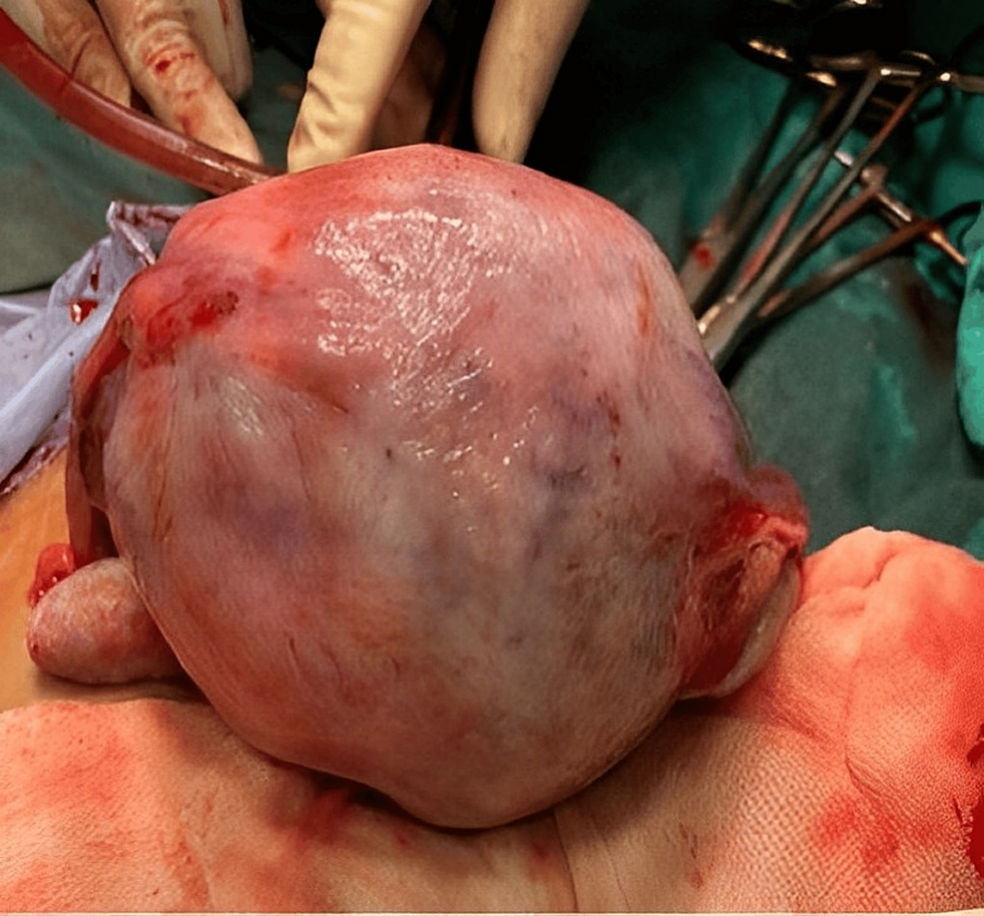
Fundal view of Couvelaire uterus with dark purple patches

The appearance of the uterus during the laparotomy remained consistent with a Couvelaire uterus, and we undertook the necessary steps to ensure hemostasis. The uterus was closed in two layers with meticulous attention to detail, ultimately achieving successful bleeding control (Figure 4).

The patient's recovery was satisfactory after the surgery, with blood product replacements administered. Her coagulation profile returned to normal, and on the third post-operative day, her haemoglobin level was measured and found to be 9.2 g/dL, indicating a positive response to treatment. She was discharged in good health three days after the surgery. Subsequent histopathological examination of the placenta confirmed the presence of retroplacental and intramembranous haemorrhage with intervillous haemorrhage, consistent with placental abruption.

## Discussion

This case illuminates an exceptionally rare complication: the presence of a Couvelaire uterus, characterized by the extensive infiltration of blood into the myometrial tissue. This condition often accompanies severe placental abruption, rendering surgical intervention a formidable challenge. Identifying this condition during the surgical procedure underscores the significance of a comprehensive intraoperative assessment and unwavering vigilance [5,6]. Effectively managing this case required the collaborative efforts of various medical specialities, including obstetricians, anesthesiologists, haematologists, and neonatologists. The cornerstone of our success lies in the timely recognition of the condition, swift surgical intervention, and the seamless coordination of post-operative care, all collectively contributing to a favourable outcome [7].

Currently, no established diagnostic clinical criterion exists for placental abruption. The New Jersey-Placental Abruption study [8] reports that the most common reasons for a clinical diagnosis of abruption include retroplacental clot(s) or bleeding (77.1%), followed by vaginal bleeding with uterine hypertonicity (27.8%) and vaginal bleeding with nonreassuring fetal status (16.1%). In our patient, a significant volume of bleeding was not observed until the time of surgery, at which point 800ml of blood loss was recorded.

Highlighting the urgency of early diagnosis in cases of placental abruption is imperative. In this instance, the delay in seeking medical attention and the tardiness in hospital booking may have exacerbated the severity of the abruption. Therefore, promoting awareness among pregnant women regarding timely prenatal care and recognising warning signs is paramount in averting such adversities [9]. Diagnostic modalities, such as trans-abdominal ultrasound, played a pivotal role in confirming the diagnosis and delineating the extent of placental abruption. Likewise, laboratory assessments, including the coagulation profile, proved invaluable in gauging the patient's bleeding risk and guiding the judicious transfusion of blood products [10].

Despite the intricacies of the case and the emergence of a Couvelaire uterus, the patient's expeditious surgical intervention and comprehensive post-operative care culminated in a favourable outcome for both the mother and the fetus. This serves as a poignant reminder of the indispensable role played by a well-coordinated healthcare team in safeguarding the lives of expectant mothers and their offspring [11].

This case report enriches the body of medical literature by shedding light on the rare complication of a Couvelaire uterus within the context of placental abruption. It underscores the imperativeness of early diagnosis and swift intervention in managing high-risk obstetric scenarios, emphasizing the need for continuous medical education and heightened awareness among healthcare providers. Furthermore, this case underscores the importance of patient education about prenatal care and recognizing warning signs during pregnancy. Healthcare systems should prioritize initiatives to augment patient awareness to mitigate delays in seeking essential medical attention.

## Conclusions

In conclusion, the placental abruption presenting with a Couvelaire uterus in our case report not only underscores the challenges and complexities faced by healthcare providers in managing obstetric emergencies but also brings to light the unique aspects and novelty of this clinical scenario. Our case offers a distinctive perspective by shedding light on the intricate interplay of factors contributing to the development of a Couvelaire uterus, including the delayed presentation of the patient and the ensuing hemoperitoneum - a complication not extensively documented in previous literature. This novel insight into the pathophysiology of a Couvelaire uterus and its association with hemoperitoneum adds to the body of knowledge surrounding this rare condition. Furthermore, our report serves as a testament to the critical role of early diagnosis, a multidisciplinary approach, and comprehensive care in achieving favourable outcomes in high-risk obstetric situations. By presenting this unique case, we aim to highlight the significance of prompt recognition and timely intervention in placental abruption cases with atypical presentations, ultimately emphasizing the critical importance of healthcare professionals in ensuring the well-being of both mothers and infants in such challenging clinical scenarios.

## References

[1] Schmidt P, Skelly CL, Raines DA (2023). Placental Abruption. https://pubmed.ncbi.nlm.nih.gov/29493960/.

[2] (2023). Placental abruption - Symptoms & Causes. Mayo Clinic. https://www.mayoclinic.org/diseases-conditions/placental-abruption/symptoms-causes/syc-20376458.

[3] Ming GS, Lee WK, Tan SQ (2020). An unusual case of placenta abruption leading to couvelaire uterus in a previable pregnancy. J Med Cases.

[4] Ophir E, Singer-Jordan J, Odeh M (2009). Abnormal placental invasion - a novel approach to treatment case report and review. Obstet Gynecol Surv.

[5] Khan S, Chughani G, Amir F, Bano K (2022). Frequency of abruptio placenta in women with pregnancy-induced hypertension. Cureus.

[6] Takeda J, Takeda S (2019). Management of disseminated intravascular coagulation associated with placental abruption and measures to improve outcomes. Obstet Gynecol Sci.

[7] Bapat R, Duran M, Piazza A (2023). A multicenter collaborative to improve postoperative pain management in the NICU. Pediatrics.

[8] Nath CA, Ananth CV, Smulian JC, Shen-Schwarz S, Kaminsky L (2007). Histologic evidence of inflammation and risk of placental abruption. Am J Obstet Gynecol.

[9] Elsasser DA, Ananth CV, Prasad V, Vintzileos AM (2010). Diagnosis of placental abruption: relationship between clinical and histopathological findings. Eur J Obstet Gynecol Reprod Biol.

[10] Shinde GR, Vaswani BP, Patange RP, Laddad MM, Bhosale RB (2016). Diagnostic performance of ultrasonography for detection of abruption and its clinical correlation and maternal and foetal outcome. J Clin Diagn Res.

[11] Parsapour H, Shafie N, Salehi AM, Assareh Z (2023). Report of a complicated case of couvelaire uterus. Case Rep Med.

